# Supplementary material for: Small but threatening: Punctate Outer Retinal Toxoplasmosis (PORT), a case series report
Source: PLoS Negl Trop Dis. 2025 Apr 11;19(4):e0013013. doi: 10.1371/journal.pntd.0013013 (PMC12058194; doi:10.1371/journal.pntd.0013013)
Supplement: Attachment — Submitted filename: PORT.docx [file pntd.0013013.s001.docx]

Comments:

I congratulate the authors on writing about a lesser-known disease manifestation of ocular Toxoplasmosis. My comments are as follows:

- Five cases in the current series are primary episodes of Ocular Toxoplasmosis. Macular punctate Toxoplasmosis (outer/inner or combination) is known to be a primary manifestation of Toxoplasmosis (OII, 2024). This needs to be highlighted in discussion as these smaller lesions are difficult to diagnose and differentiate leading to delayed diagnosis of Toxoplasmosis.
- How many of the cases have the concurrent presence of PIRT and PORT lesions at first visits?
- Did all the cases develop full-thickness retinitis on follow up at the site of the PORT lesions?
- I urge the authors to correct a few spelling errors in the manuscript. (ex. Line 157, 195).
