# Supplementary material for: Small but threatening: Punctate Outer Retinal Toxoplasmosis (PORT), a case series report
Source: PLoS Negl Trop Dis. 2025 Apr 11;19(4):e0013013. doi: 10.1371/journal.pntd.0013013 (PMC12058194; doi:10.1371/journal.pntd.0013013)
Supplement: Attachment — Submitted filename: Comments to the Authors JAN23.docx [file pntd.0013013.s003.docx]

**Comments to the Authors:**

**Reviewers' Comments:**

Reviewer's Responses to Questions

**Key Review Criteria Required for Acceptance?**

As you describe the new analyses required for acceptance, please consider the following:

**Methods**

Reviewer #1: (No Response)

Reviewer #2: This is a case series reporting the clinical and imaging features of patients affected by punctate outer retina toxoplasmosis (PORT). The study design is appropriate and the clinical cases are well presented.

Response: Thank for your positive comments.

Reviewer #3: Punctate outer retinal toxoplasmosis is considered a forme froste form of Ocular Toxoplasmosis. How many of the total eyes develop full thickness typical retinitis during the follow up and what was the interval between these?

Response: Three out of eight patients (patients 5, 7 and 8) in our series presented full thickness retinochoroidal scars alongside active PORT lesions. However, these three patients weren’t known in our hospital priorly in order to be able to document time intervals between classical lesions (seen at first-time visit as scars) and PORT lesions. Patient 2 presented two PORT activations, the second episode occurred 2 months after the first one, yet no full thickness typical retinitis was documented in this patient. None of our patients presented full thickness typical retinitis after PORT manifestation.

One of the ways to check timeline of infection to formation of these lesions is Toxoplasma IgG avidity. Was it done in these cases?

Response: We know that IgG avidity test provides information about time of exposure to Toxoplasma. It can complement diagnosis in different phases of disease.

We did not perform avidity test in any patient. All patients had Toxoplasma Ig G with a median of 274.1 IU/dL (IQR 349 75.25 – 1293). Seven out of eight patients (87.5%) had negative IgM and significantly elevated 350 IgG, supporting OT diagnosis.

The detection of high-avidity antibodies effectively excludes infection acquired within the past 3–4 months; however, low-avidity antibodies may remain detectable for more than 3 months after infection. (Montoya KG et al Lancet 2004; 363: 1965–76)

Also described by Kalogeropoulos D (Int Ophthalmol 2022;42:295–321) avidity test has limitations, including the value to determine as high and low result, making other serologic studies required in borderline avidity.

**Results**

Reviewer #1: (No Response)

Reviewer #2: The clinical results are clearly presented. The images are appropriate and present well the clinical pictures

Response: Thank for your observations.

Reviewer #3: Macular punctate toxoplasmosis is the primary manifestation of ocular Toxoplasmosis - How many cases in the current series satisfy this criterion?

Response: 5 patients were diagnosed as a first episode. It is described on Table 1.

Did any of the cases have additional punctate inner retinal lesions as the combination of these lesions PIRT and PORT is a known manifestation.

Response: Eight out of eight patients presented PORT in macular region. No PORT lesions were discovered in peripheral retina. Four patients presented PORT in the fovea (patients 1, 2, 3 and 4), two patients had perifoveal PORT lesions (patients 6 and 8) and two of them presented PORT outside foveal area but within the macula (patients 5 and 7). This was added in discussion.

**Conclusions**

Reviewer #1: (No Response)

Reviewer #2: The conclusions are well supported. However, electrophysiological testing including multifocal electroretinography could have been very informative either for diagnosis or the follow-up after treatment.

Response: Thanks for the observation. A paragraph was added related to ERG.

We added this paragraph in in the Discussion; “The role of both flash electroretinogram (FERG) and multifocal flash electroretinogram (mfERG) have been also studied while assessing patients with OT. Depending on the site of the lesion, mfERG could demonstrate a wide variety of alterations, this could go from local changes corresponding to the patient’s scotoma in visual field examination; to selective loss of responses within the central 10 degrees. Norose et al. investigated the use of FERG in mice infected with OT, they concluded that flash ERG can detect site of infection, predict virulence of the organism and therefore predict the patient’s prognosis. Norose Up to this date, there is no study that investigates the role of retinal electrophysiology in PORT. We believe FERG and mfERG could be useful in patients with suspected OT that present severe vitritis when diagnosis is not clear. However, PORT is characterized by having no or mild vitritis, therefore we don’t generally perform these tests in our hospital in this circumstances.”

Reviewer #3: Primary ocular Toxoplasmosis without old scar is often a risk factor for atypical presentation - this needs to be highlighted in the discussion section.

Response: Atypical presentations can indeed be detected in patients with or without old OT scars. However, to our knowledge, not having old scars has not been yet categorized as a risk factor by itself.

**Editorial and Data Presentation Modifications?**

Reviewer #1: (No Response)

Reviewer #2: Minor revisions include a discussion on the role of retinal electrophysiology, especially multifocal ERG, in the management of PORT.

Response: Thanks for the valuable and additional information. We included mERG in the discussion.

Reviewer #3: Minor revision suggested

**Summary and General Comments**

Reviewer #1: (No Response)

Reviewer #2: The study is interesting and improve the knowledge in the field. I recommend minor changes.

Response: We checked comments and made them

Reviewer #3: None

**Figure resubmission:**
While revising your submission, please upload your figure files to the Preflight Analysis and Conversion Engine (PACE) digital diagnostic tool, <https://pacev2.apexcovantage.com/>. PACE helps ensure that figures meet PLOS requirements. To use PACE, you must first register as a user. Registration is free. Then, login and navigate to the UPLOAD tab, where you will find detailed instructions on how to use the tool. If you encounter any issues or have any questions when using PACE, please email PLOS at [figures@plos.org](mailto:figures@plos.org). Please note that Supporting Information files do not need this step. If there are other versions of figure files still present in your submission file inventory at resubmission, please replace them with the PACE-processed versions.

Response: Images were uploaded in PACE

**Reproducibility:**
To enhance the reproducibility of your results, we recommend that authors of applicable studies deposit laboratory protocols in [protocols.io](http://protocols.io), where a protocol can be assigned its own identifier (DOI) such that it can be cited independently in the future. Additionally, PLOS ONE offers an option to publish peer-reviewed clinical study protocols. Read more information on sharing protocols at <https://plos.org/protocols?utm_medium=editorial-email&utm_source=authorletters&utm_campaign=protocols>
