# Supplementary material for: Small but threatening: Punctate Outer Retinal Toxoplasmosis (PORT), a case series report
Source: PLoS Negl Trop Dis. 2025 Apr 11;19(4):e0013013. doi: 10.1371/journal.pntd.0013013 (PMC12058194; doi:10.1371/journal.pntd.0013013)
Supplement: Attachment — Submitted filename: Response to reviewers 180325.docx [file pntd.0013013.s004.docx]

**Reviewers' comments:**

Reviewer's Responses to Questions

We thank for your observations and address each one of them.

**Methods**

Reviewer #1: (No Response)

Reviewer #2: The study objectives are clearly reported, the study design is appropriate, the population clearly described and the sample size sufficient. Statistical analysis is correct. No ethical concerns.

Reviewer #3: The reviewer’s queries were answered adequately.

**Results**

Reviewer #1: (No Response)

Reviewer #2: The analysis marches the analysis plan. The results are clearly presented. Good quality of figures.

Reviewer #3: The reviewer’s queries were answered adequately.

**Conclusions**

Reviewer #1: (No Response)

Reviewer #2: The conclusions are supported by the data and the limitations are reported. The study is novel and of public health relevance.

Reviewer #3: The reviewer’s queries were answered adequately.

**Editorial and Data Presentation Modifications**

Reviewer #1: (No Response)

Reviewer #2: I suggest to add some comments to the discussion regarding the differential diagnosis with inherited macular dystrophies that can manifest with unilateral lesions i.e. RP1L1 gene-related macular dystrophies.

**Response**. Thanks for the observation. We added a paragraph of differential diagnosis with clinical, ERG and OCT studies.

“OCT alterations seen in PORT could resemble some macular diseases, such as RP1L1-pathogenic-variant-related occult macular dystrophy (OMD) (16). The most common findings in SD-OCT include disruptions or absence of the ellipsoid zone (EZ) and the interdigitation zone (IZ) (17), along with attenuation of RPE in the macular area (18). However, OMD is characterized by a normal fundus examination (17), hence its name “occult”, and it typically presents as a bilateral alteration (18, 19, 20). The differential diagnosis of these conditions relies on these characteristics, as in PORT 1) the classical yellowish-white lesion is visible in fundus examination, 2) this lesion can be found outside the macular area, 3) bilateral involvement is less common. Additionally, OMD tends to be a progressive disease, whereas in PORT, visual acuity decreases more abruptly due to acute inflammation, and 4) changes in multifocal electroretinogram in OMD are always confined to the macular area (21), while in PORT, changes vary depending on where the lesion is located.”

Reviewer #3: None

**Summary and General Comments**
Use this section to provide overall comments, discuss strengths/weaknesses of the study, novelty, significance, general execution and scholarship. You may also include additional comments for the author, including concerns about dual publication, research ethics, or publication ethics. If requesting major revision, please articulate the new experiments that are needed.

Reviewer #1: (No Response)

Reviewer #2: This is an interesting case series describing the lesions of punctate outer retina toxoplasmosis. The clinical pictures presented in the study sometimes may be reminiscent of inherited retinal dystrophies that may not be necessarily bilateral and symmetric (see for example those related to mutations in the RP1L1 gene). The authors should discuss in more detail the differential diagnosis with inherited dystrophies.

**Response**: Thank you for your comment. We appreciate your observation regarding the resemblance of PORT to inherited retinal dystrophies, including those associated with RP1L1 mutations. We acknowledge that certain inherited dystrophies may present unilaterally or asymmetrically, further complicating differential diagnosis. To address this, expanded our discussion by comparing the key clinical, imaging, and electrophysiological characteristics.

Reviewer #3: The article could be accepted
